# Tannin-Based Hybrid Materials and Their Applications: A Review

**DOI:** 10.3390/molecules25214910

**Published:** 2020-10-23

**Authors:** Ann-Kathrin Koopmann, Christian Schuster, Jorge Torres-Rodríguez, Stefan Kain, Heidi Pertl-Obermeyer, Alexander Petutschnigg, Nicola Hüsing

**Affiliations:** 1 Salzburg Center for Smart Materials, Jakob-Haringer-Straße 2a, 5020 Salzburg, Austria; ann-kathrin.koopmann@sbg.ac.at (A.-K.K.); christian.schuster@sbg.ac.at (C.S.); jorge.torres@sbg.ac.at (J.T.-R.); stefan.kain@fh-salzburg.ac.at (S.K.); adelheid.pertl-obermeyer@sbg.ac.at (H.P.-O.); alexander.petutschnigg@fh-salzburg.ac.at (A.P.); 2 Department of Chemistry and Physics of Materials, Paris-Lodron-University Salzburg, Jakob-Haringer-Straße 2A, 5020 Salzburg, Austria; 3 Forest Products Technology & Timber Constructions Department, Salzburg University of Applied Sciences, Markt 136a, 5431 Kuchl, Austria

**Keywords:** tannins, polyphenolic molecule, sustainability, hybrids, green chemistry

## Abstract

Tannins are eco-friendly, bio-sourced, natural, and highly reactive polyphenols. In the past decades, the understanding of their versatile properties has grown substantially alongside a continuously broadening of the tannins’ application scope. In particular, recently, tannins have been increasingly investigated for their interaction with other species in order to obtain tannin-based hybrid systems that feature advanced and/or novel properties. Furthermore, in virtue of the tannins’ chemistry and their high reactivity, they either physicochemically or physically interact with a wide variety of different compounds, including metals and ceramics, as well as a number of organic species. Such hybrid or hybrid-like systems allow the preparation of various advanced nanomaterials, featuring improved performances compared to the current ones. Consequently, these diverse-shaped materials have potential use in wastewater treatment or catalysis, as well as in some novel fields such as UV-shielding, functional food packaging, and biomedicine. Since these kinds of tannin-based hybrids represent an emerging field, thus far no comprehensive overview concerning their potential as functional chemical building blocks is available. Hence, this review aims to provide a structured summary of the current state of research regarding tannin-based hybrids, detailed findings on the chemical mechanisms as well as their fields of application.

## 1. Introduction

The plant-based polyphenolic tannins are the fourth most abundant form of terrestrial biomass extracted compounds right after cellulose, hemicellulose, and lignin [1]. Furthermore, tannin presents after lignin the most prevalent source of natural aromatic macromolecules with a global industrial production of over 160,000 tons/year [2,3].

Tannins are present in different ratios in vascular plants, in which they function as defense agents against herbivores, fungi, and microorganisms. Furthermore, they are also present in non-vascular plants, i.e. algae, in which they have various metabolic roles [3]. In contrast to lignin, tannins are predominantly present in the soft tissue of plants, such as needles, leaves, or bark [3]. Generally, the highest concentration of tannin is found within the tree barks of pine (*Pinus* sp.), wattle/mimosa (*Acacia mearnsii*), or oak (*Quercus* sp.) trees [4,5]. Furthermore, tannin can be found in the gambier (*Uncaria gambier*) sheets or the wood of the chestnut (*Castanea* sp.) or quebracho (*Schinopsis* sp.) [3].

The term “tannin” is derived from the tanning process, whereby animal skin is converted into the leather using aqueous plant extracts [6]. The tanning industry in the twentieth century depicts the first industrial applications of tannins and thus coined the term of the polyphenolic, natural wood extract tannin [5,7]. However, over the last past decades, the range of applications has been tremendously broadened, for example towards the fields of oenology, medicine, and materials engineering [8,9,10,11]. 

**Figure 1:**
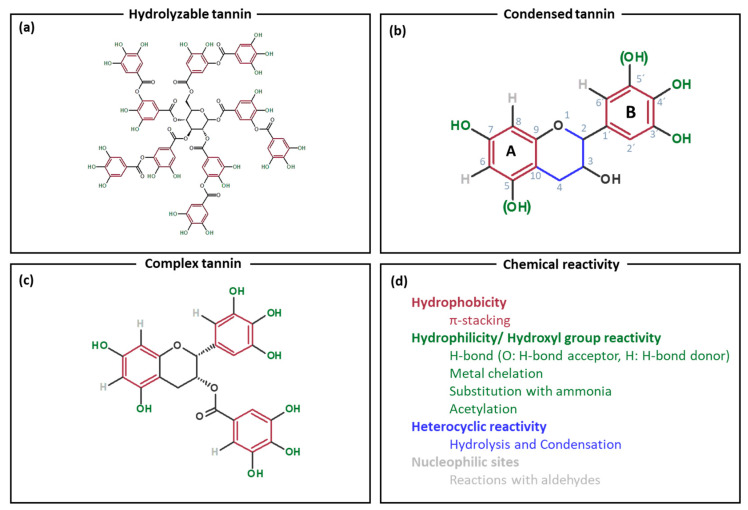
Example structures for different classes of tannins. (**a**) Structure of a tannic acid (TA) belonging to the hydrolysable tannins. (**b**) The monomeric flavonoid unit of condensed tannins which is usually connected to adjacent units at positions 4 and 6 or 8. (**c**) Epigallocatechin-gallate as an example for complex tannins. (**d**) Most important chemical reactivity properties of tannins color-coded in accordance with the respectively involved functional groups or moieties.

Tannins can be classified based on their chemical structure into two main conventional groups, namely hydrolyzable and condensed tannins [10,12]. Figure 1 schematically depicts the different tannin classes as well as their chemical reactivity. 

Hydrolyzable tannins, with tannic acid (Figure 1a) as the most prominent example, are further subdivided into two groups, the gallo- as well as ellagitannins. Gallotannins form gallic acid as well as its derivatives during hydrolysis. On the contrary, the so-called ellagitannins produce ellagic acid after hydrolysis. As already adverted by its name, this class of tannins can be easily hydrolyzed by weak bases or acids [9,10]. The main application field of hydrolyzable tannins is in the tanning industry. However, they solely exhibit a limited availability as they only make up less than 10% of the worldwide commercial tannin production, and hence high costs are involved for the production of hydrolyzable tannins [3]. 

Condensed tannins represent the most abundant class of tannins, which makes up more than 90% of the worldwide commercial tannin production. Condensed tannins are oligomers and polymers of flavonoid units (Figure 1b), whereby 3–8 flavonoid repetition units are needed in order to denote a compound as condensed tannin [3,9]. Each monoflavonoid unit consists of two phenolic rings (A- and B-ring) with different reactivity. The A-ring exhibits more reactive nucleophilic centers (located activation of A-ring at C6 and C8) in contrast to the B-ring, based on differences in the location of the hydroxyl groups at these rings. There are two configurations for each phenolic ring as the hydroxyl group at positions 5 and 5′ can be present or absent [3]. Thus, condensed tannins (also called proanthocyanidins) are made up of four different main building blocks: profisetidin, procyanidin, prorobinetinidin, and prodelphinidin. Depending on the tannin source, one of these species is predominantly available in the extract, e.g., probinetinidin is prevalent in mimosa extracts and profisetidin in quebracho extracts [2,13]. Oxidative coupling between the monomeric flavonoid units of the tannin occurs commonly between the C4 and C6 or C4 and C8 positions. More precise, in profisetidins and prorobinetinidins the flavanoidunits are most likely connected through C4-C6 bonds, whereas procyanidins and prodelphinidins are predominantly linked by C4-C8 bonds [3,10,14]. Furthermore, the degree of polymerization varies between the different tannin extracts as mimosa and quebracho tannin show a high degree of polymerization, whereas pine and gambier only feature a medium and low degree of polymerization, respectively [2]. 

Complex tannins were discovered as an additional group of tannins back in 1985, which consist of an ellagitannin unit as well as of a flavanoidunit, resulting in sophisticated structures. First, this tannin class was labeled as “non-classified” tannins and later the term “complex tannins” was introduced. However, due to their complex structure and relatively low abundance, this class of tannin finds limited adaptation in tannin based applications [10].

Apart from these conventional tannins, there are also non-vascular plant (e.g., algae) tannins, so-called phlorotannins, which are generated from the polymerization of phloroglucinol [3,15]. However, this class of tannins is not discussed in this review. 

Due to their multiple aliphatic hydroxyls and phenolic hydroxyl groups, tannins have the ability to form complexes with proteins and polysaccharides as well as to interact with inorganic salts [12,14,16]. In general, the significant tendency of tannins to adsorb and reduce metal ions and hence generate stable metal complexes has to be stressed [17,18,19]. Particularly, condensed tannins possess a higher reactivity compared to hydrolyzable tannins and therefore are often chemically and economically of higher interest [19,20]. In addition, condensed tannins also show a heterocyclic reactivity as well as a reactivity of the aromatic nucleophilic sites. The highly reactive phenolic hydroxyl groups facilitate chemical modifications such as substitution with ammonia [21,22,23], acetylation [24,25], or reaction with epoxy groups [26]. Furthermore, electrophilic substitution at the nucleophilic sites can occur, which allows reactions such as bromination or reactions with aldehydes. Besides, condensed tannins are prone to feature a heterocyclic reactivity as they undergo hydrolysis (degradation) and autocondensation reactions (heterocyclic ring opening and subsequent nucleophilic addition to another tannin unit) in catalyzed conditions [3]. Moreover, non-covalent π-π interactions can occur between the tannins’ aromatic rings and other aromatic systems, such as graphene oxide [27]. However, the chemical reactivity of condensed tannins is discussed in greater detail elsewhere [3,6,9].

Due to the high reactivity of tannins, they are prone to interact with other molecules, and hence recently multicomponent materials, partly made of the sustainable tannin, have evoked increased interest. More precisely, in the last two decades, tannin-derived hybrid materials are an emerging field, as they feature new perspectives due to combined or enhanced materials’ properties and hence allow an expansion of the materials’ application scope [28,29]. In particular, the interaction of inorganic and organic materials, in order to generate new materials with favorable properties of each organic and inorganics, is discussed [29,30]. The addition of an inorganic component, such as silica, to tannin can increase the thermal, and chemical properties of the tannin-derived materials allowing to provoke improved outcomes for diverse applications [31,32]. Furthermore, another reason for the increasing popularity of tannin-derived hybrid materials is the hydrophilicity and hence the water-solubility of pure tannins, which limits their use for certain applications, such as water purification [33,34]. Therefore, immobilization onto water-insoluble matrices, such as silicates, cellulose, and alginate [31,32,34,35,36], or the reaction of tannins’ phenolic hydroxyl groups with metal ions to form insoluble tannin–metal hybrids [37] is required in order to permit their use in these kinds of applications. Apart from water-treatment, which is the most frequently studied application, the increasingly studied field of tannin-derived hybrid materials has evoked possible new applications, such as photocatalysis as well as biomedical and stimuli-responsive materials. However, research is currently ongoing within the scientific community, to even further explore and expand the applicability of tannin-derived hybrid materials.

Therefore, within this review, the recent attempts to generate tannin-derived hybrid materials are summarized in order to provide a comprehensive overview. More precisely, tannin–metal, tannin–ceramic, and tannin–polymer hybrids are discussed in detail, with a particular focus on their chemical structure, material properties, and possible fields of application. Conclusively, emerging applications are displayed and future perspectives are described.

## 2. Hybrid Materials

Hybrid materials typically combine appreciably different compounds into a single entity and often yield properties superior to those expected from the simple sum of its parts. This emergence of new and useful properties depends on the length-scale of mixing as well as the type of constituent used as the companion to the other. Commonly, for the combination with tannins, the latter would be expected to be an inorganic material to be labeled as a hybrid. However, new and useful properties can also emerge from combinations with organic materials permitting their classification as hybrid-like materials. As shown in the following sections, tannins possess a unique set of properties that allow their combination and interaction with a wide variety of compounds resulting in materials with intriguing properties. Nevertheless, the scientific community has begun to recognize this potential recently. That is to say, tannin-derived hybrid (or hybrid-like) materials based on both inorganic and organic counterparts have been described in the literature to some extent, yet the field is relatively new, unstructured, and unexplored regarding the reaction chemistry as well as potential fields of application. Therefore, the following sections give a structured and comprehensive summary of materials obtainable by the hybridization of tannins with metallic, ceramic, and organic counterparts, the involved chemistry, and their potential applications.

### 2.1. Tannin–Metal Hybrids

Tannins, which are prone to metal chelation, are widely applied in the field of metal adsorption, in particular for water and wastewater treatment as well as for the recovery of valuable metals from a fluid. In the process of metal chelation, especially the ortho-dihydroxyl groups of the tannins’ catechol ring (3′-4′ site) represent the most involved phenolic hydroxyl groups, which hence allow catechol-like coordination with metals [18,19,38]. However, transition metals can also form coordination complexes to the polyphenol not solely via its orthodihydroxy (catechol) but also via its trihydroxybenzene (pyrogallol) moieties, if a hydroxyl group at the 5′ site is present [39,40]. The metal chelation process takes place for all classes of tannins; however, condensed tannins are most likely to form complexes with metals due to their high chemical reactivity in comparison to hydrolyzable tannins [19]. 

**Figure 2:**
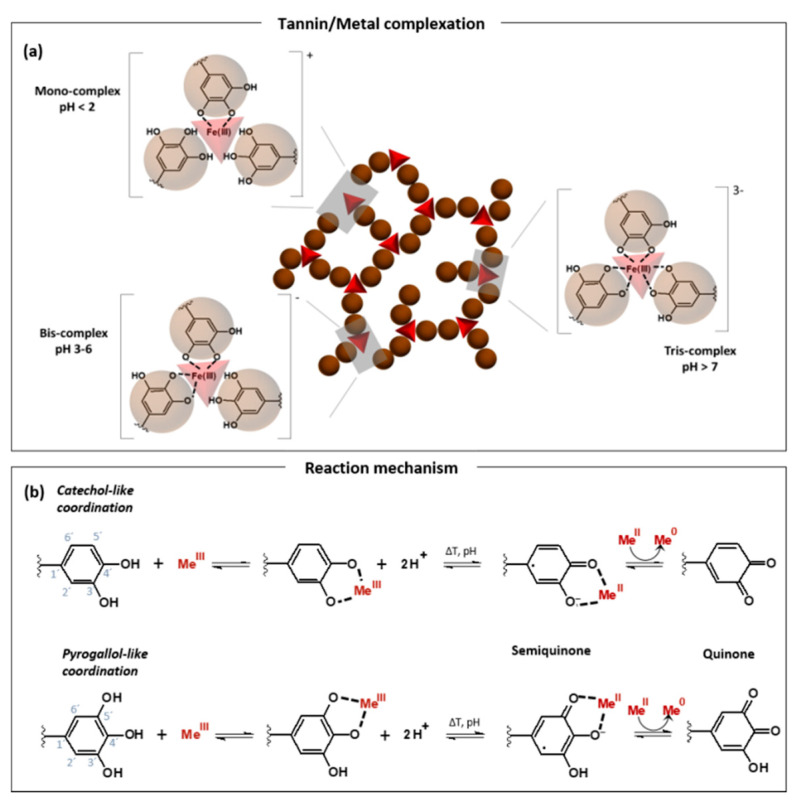
Metal complexation and reduction behavior and involved mechanisms. (**a**) Schematic representation of the formation of a metal–phenolic network between tannin and iron ions where, depending on the pH value, a mono-, bis- or tris-complex is formed, and the network formation capability is accordingly changed. (**b**) General reaction mechanisms for the complexation of metal ions and the subsequent ligand to metal electron transfer that enables tannins to reduce metal ions to elemental metal. The reduction progresses from the fully reduced forms through a semiquinoid form with one carbonyl to a quinoid form with two carbonyl groups.

In general, tannins form metal chelation complexes with a broad variety of metal ions, whereby the tannin functions as a reducing as well as stabilizing agent [40,41]. Furthermore, the tannin–metal complex formation is significantly influenced by the initial pH value [38]. Figure 2 schematically depicts the complexation behavior of tannins with metals, precisely for iron, which is the most frequently studied metal ion regarding the complexation behavior with tannins. A tannin–metal mono-complex is formed at pH below 2, whereas a pH of 3–6 gives a bis-complex, and a stable tris-complex is generated at a pH of above 7 [42,43]. Other metal species also show such pH-dependent complex formation, but the exact pH ranges may vary. However, these dependencies have not been studied in great detail within the literature so far. 

Regarding water treatment and in order to provide clean water without contaminants, it is important to efficiently remove toxic metal contaminations, such as copper, lead, or cadmium, which can possess serious health risks to living organisms [9]. Current methods of water treatment, e.g., electrochemical methods, imply high cost as well often insufficient adsorbent capacities of the toxic metals [9,44], and hence the usage of adsorbent materials has been thoroughly investigated. In particular, the usage of the low-cost, natural tannins has been widely investigated for their ability to remove lead [35,45,46,47], copper [35,46,47], cadmium [46], chromium [35,48,49,50,51], nickel [46], and zinc [46,52] from wastewater. Furthermore, several studies have been conducted regarding the recovery of precious metals from liquids. In the literature, it has been shown that tannin-based adsorbent materials possess the ability to regain gold [53,54,55,56,57,58,59,60], silver [61], and palladium [53,54,55,57,58,61,62] ions from aqueous solutions. The recent research progress on tannin-based adsorbent materials can be found in greater detail elsewhere [19].

#### 2.1.1. Tannin–Iron Hybrids

To increase the adsorbent capacity for all metals and especially for those that only show minor adsorption with unmodified tannin materials, metal-loading (mainly iron) of tannins has been suggested. Accordingly, a wattle tannin-based gel network forms by the polymerization reaction with formaldehyde, which acts as a crosslinker. The subsequent oxidation treatment using concentrated nitric acid induces the oxidation of the tannins’ hydroxyl groups to a carbonyl group, which generates a mono-type complex of the oxidized tannin gel and Fe(III). More precisely, the oxidation treatment achieved an increase of 2.3 in the uptake of iron (8.5 wt% of iron in the oxidized tannin gel) compared to the untreated tannin gel. Furthermore, the study in [17] depicts that the non-iron-doped tannin as well as the oxidized tannin gel does not possess any ability to adsorb phosphate from aqueous solutions. In contrast to that, it is found that the iron-doped oxidized tannin gel features an adsorption selectivity for phosphate over other anionic species (chloride, nitrate, and sulfate ions) as well as a pH independency of the adsorption capacity in the range of initial pH of 3–12.

More recently, in 2017, Luo et al. [63] presented a method to generate a lead (Pb^2+^) and mercury (Hg^2+^), which are highly toxic and carcinogenic heavy metals, adsorbent material based on tannic acid (hydrolyzable tannin) modified iron oxide core–shell nanoparticles (Fe_3_O_4_@TA-Fe^3+^). In comparison to the research before, TA does not need an additional oxidation treatment as it already forms a stable octahedral complex by the reaction of three tannic acids’ galloyl groups with each Fe^3+^ ion, resulting in a crosslinked TA-Fe^3+^ film [43,63]. To generate such stable Fe_3_O_4_@TA-Fe^3+^ complex, magnetite nanoparticles, ferric chloride, tannic acid, and water are added in a defined ratio [63]. The generated complex permits the adsorption of Pb^2+^ and Hg^2+^ with increased adsorption capacities, compared to previously trialed non-iron-doped tannin-based adsorbent materials for Pb^2+^ [35,45,46,47] and Hg^2+^ [64]. Additional literature concerning the iron oxide nanoparticles used as a support in the above-mentioned materials is further discussed in Section 2.2 within the tannin–ceramic hybrid systems.

Despite the functionality of tannin–metal hybrids as adsorbent materials, they are also discussed for other applications, for example biomedical ones including drug-delivery agents in organisms [42]. By adhering to the reaction of tannin and iron, Bartzoka et al. [42], for instance, trialed all different classes of tannins regarding their formation of microcapsules with iron(III). More precisely, iron–polyphenol coordination complexes are generated by mixing an aqueous solution of FeCl_3_ with a hydrolyzable tannin (tannic acid), a complex tannin (epigallocatechin-3-O-gallate), or a condensed tannin (*Acacia mearnsii* or *Schinopsis balansae*) at pH 7.4 and subsequent ultrasonic treatment [42]. Thereby, the tannins’ inherent catechol groups function as bidentate iron chelators, allowing octahedral iron complexes to be formed, which however feature different structures depending on the initial pH value, as described above [42,43]. However, since condensed tannins contain in general a higher amount of catecholic groups, they form complexes more readily compared to hydrolyzable and complex tannins, which results in a lower average degree of polymerization. Thus, solely the addition of metals ensures the stable microcapsule assembly of monomeric tannins. These ferric hybrid microcapsules allow working as an active delivery system against *M. tuberculosis*, when loaded with an anti-tuberculosis agent [42]. 

#### 2.1.2. Tannin–Noble Metal Hybrids

Apart from iron, also other metal ions have been investigated for their suitability to form tannin–metal hybrids. For example, the generation of stable silver nanoparticles can be carried out by mixing AgNO_3_ and tannic acid, which functions as a reducing as well as a stabilizing agent of the respective metal ions [41,65,66]. Tannic acid stabilized silver nanoparticles feature an antimicrobial activity as well as electrical conductivity and can be used when immobilized to clays for electrochemical studies [41], as an antileishmanial active agent against *Leishmania* (a severe parasitic infection) [65], or as an antiviral agent against the Herpes Simplex Virus Infection-Type 2 [66].

Likewise, tannic acid stabilized/coated gold nanoparticles are prepared by mixing a chloroauric acid solution with tannic acid. Herein, tannic acid functions as a stabilizer but also allows the reduction of Au^3+^ to form Au nanoparticles [65,67]. Furthermore, it is shown that tannin–gold hybrid materials cannot solely be produced using hydrolyzable tannins but also in a similar manner in a one-pot approach by using the condensed bayberry tannin [68]. The third class of tannins (complex tannins) are also able to generate tannin–gold hybrids such as epigallocatechin-gallate (EGCg) functionalized radioactive gold nanoparticles, which are prepared by mixing EGCg and radioactive tetrachloroauric acid (H^198^AuCl_4_) and can function as a prostate cancer diagnostic as well as a therapeutic agent [69].

Recently, Park et al. [70] demonstrated that the formulation of a complex tannin–metal hybrid such as TA-Cu(II) is feasible for applications in photoresponsive drug delivery carriers. In detail, prefilled (with rhodamine B and a photoacid generator) mesoporous silica nanoparticles (MSNs) can be covered with a pH-sensitive shell composed by coordinated TA-Cu(II) complexes. Under certain conditions, the mesoporosity of the MSNs is blocked by the TA-Cu complexes by ionic interactions. Thus, upon exposure to UV radiation, the photoacid generatortriggers local pH variations, which, in turn, decompose the TA-Cu(II)-blocking-shell, resulting in the release of therhodamine B. Certainly, this interesting advance creates new possibilities for applications of tannin–metal hybrids in medical applications.

#### 2.1.3. Tannin-Based Metal–Carbon Hybrids

Furthermore, the hydrothermal treatment of tannin–metal hybrid materials recently became of interest, resulting in metal–carbon materials. Braghiroli et al. [71] trialed the usage of condensed mimosa tannin combined with various dissolved transition metals (V, Cr, Ni, and Fe) as well as their hydrothermal treatment for the generation of porous metal oxides and metal–carbon hybrid materials. On the one hand, they investigated the influence of the four above-mentioned metals, which are dissolved in the aqueous tannin solution, then carbonized at 180 °C for 24 h, and subsequently calcined at 550 °C in air, on the morphology of the resulting particles. Significant differences are observed in the chelation behavior of V and Cr in contrast to Ni and Fe with tannin. The metals V and Cr were, after hydrothermal treatment, highly dispersed in the carbonaceous structure, implying that these metals chelated throughout the entire tannin-based polymer network. Hence, the resultant porous oxides feature a nanoparticle morphology in the case of V and Cr, whereas Ni and Fe produce a hollow microsphere porous metal oxide structure, in which these metals only concentrate in the outer part of the microspheres. It is assumed that these different morphologies arose from the diverse complexation manner of the metal ions with tannins. Moreover, an iron/carbon hybrid material, which is comprised of iron and magnetite dispersed in graphitic carbon, is generated by the pyrolysis of iron-doped carbonaceous particles at 900 °C in an inert atmosphere. The resultant material is discussed to function as an adsorbent for decontamination purposes; however, an additional activation step is required in order to improve the materials’ textual properties (e.g., increase specific surface area) and convert them into magnetic-activated carbons [71]. 

Furthermore, the same group investigated the introduction of silver nanoparticles into a carbonaceous shell [72]. Therefore, condensed tannin is thermally treated in an autoclave for 24 h at 180 °C and, subsequently, a silver nitrate solution is added to the autoclave followed by a carbonization step (900 °C, 3 h, Ni-atmosphere). In general, tannin causes the silver ions to be reduced in order to generate a carbonaceous material with entrapped silver nanoparticles, which is interesting for various applications in the field of biochemistry as well as biosensors [72]. 

However, hydrolyzable tannin-derived metal hybrids are also examined for the suitability to produce carbon hybrid materials [39,73]. For example, iron(III) chloride hexahydrate and tannic acid form a TA-Fe coordination complex and can function as a precursor for porous carbons, which, after an activation step with KOH to increase porosity, seems to be an adequate candidate for CO_2_ capture and storage [39]. Similarly, iron(III) nitrate nonahydrate and TA also form a coordination complex, which can be used as a precursor for Fe/N-doped carbon hybrids [73]. This kind of iron–nitrogen clusters embedded into porous N-doped carbons function as Fenton reagent, which represents a catalyst that oxidizes contaminants and wastewaters and is used in batteries, supercapacitors, or fuel cells [73].

Overall, it can be summarized that all different classes of tannins form coordination complexes with various metal ions under certain reaction conditions. These resulting hybrid materials are often used as an adsorbent for a range of reactive species. However, apart from water treatment, during the past decade, tannin–metal hybrid materials have also been investigated for their suitability for biomedical as well as electrochemical applications.

### 2.2. Tannin–Ceramic Hybrids

**Figure 3:**
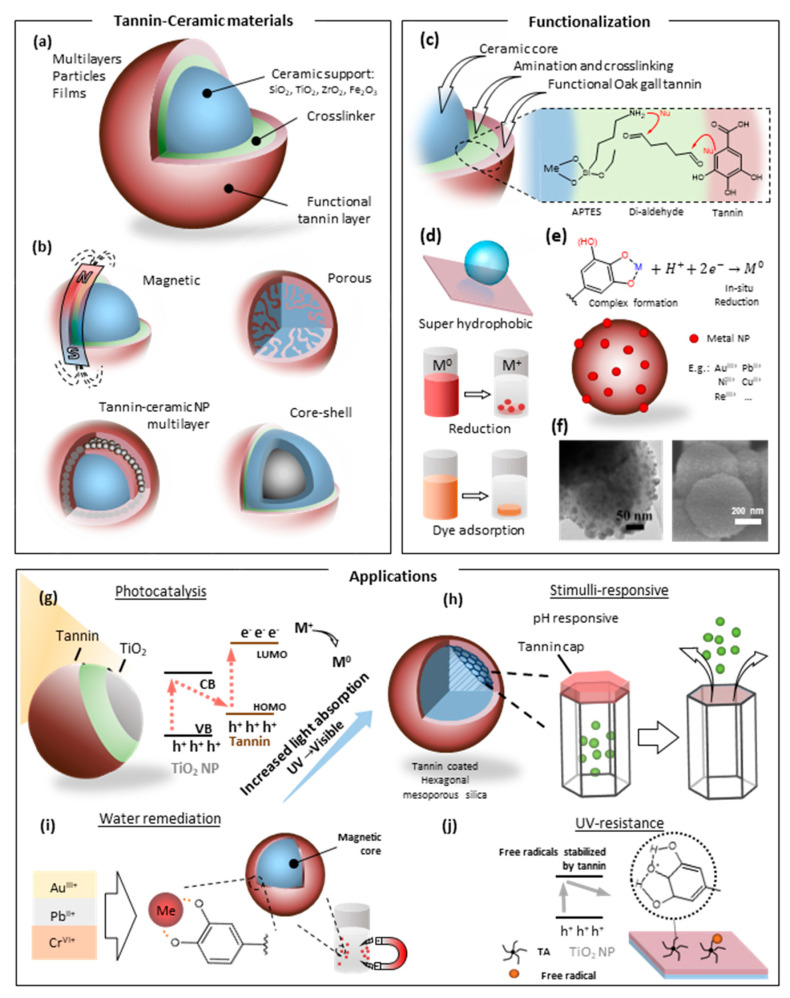
Overview of tannin–ceramic materials. (**a**) Schematic representation of the typical layout of hybrid tannin–ceramic particles. (**b**) Multiple frequently encountered architectures where the ceramic and tannin parts can have several morphologies and structural arrangements. (**c**) Schematic representation of the ceramic surface functionalization driven by the physicochemical interactions of active groups on the surface of the ceramic core, of the cross-linker and the tannin to form tannin–ceramic hybrids. (**d**) Characteristics and applications of TA-APTES coatings, which are further functionalized with various different reactants, applied to multiple substrates [76]. (**e**) Net reaction on the surface of a tannin-based hybrid material for in-situ metal-ion scavenging and redox reaction to form ceramic–tannin–metal systems shown in (**f**). (**f**) Microstructure of core–shell hybrid particles with deposited metal nanoparticles as the result of in-situ redox reactions. Adapted with permission from Ref. [77]. Copyright 2018 Elsevier Inc. (**g**–**j**) Schematic representations of example applications of tannin–ceramic hybrid materials. (**g**) TiO_2_–tannin hybrids with enhanced photocatalytic metal ion reduction activity and the suggested Z-scheme of the net electron pathway [78]. (**h**) pH-responsive release of drugs from the hexagonal cavities of mesoporous particles capped with a tannin-based coating. Adapted from Ref. [79]. (**i**) Advanced water remediation by metal ion adsorption to magnetic tannin–ceramic hybrid particles and subsequent magnetic collection. Adapted from Ref. [80]. (**j**) Mechanism of enhanced UV-resistance by the tannin-mediated radical stabilization [81].

As mentioned in the previous section, within the range of applications of tannin-based materials, the recovery of high-value metals, as well as the remediation of aqueous effluents from harmful heavy metals is of great interest to the scientific community [74]. This is due to the potential that tannins naturally allow reducing metal ions or form chelates very easily [75]. This is especially favorable for the treatment of different water sources polluted with toxic metals such as Cr^3+^, Hg^2+^, Cd^2+^, and Pb^2+^, among others. In addition, the recovery of precious metals such as Au^3+^, Pt^2+^, Ag^+^, etc. from aqueous production effluents represents a point of interest [32]. In this context, to take advantage of the capacities of the tannin as a metal-sorbent component in aqueous suspensions, it is first necessary to tackle its water solubility. Regarding tannin–ceramic hybrids, the most studied approach is the immobilization of tannin compounds onto a mesoporous structure (e.g., SiO_2_, TiO_2_, ZrO_2_, Al_2_O_3_, etc.), thus merging the outstanding metal-affinity of tannins with the robustness and large surface area of the ceramic matrix. In this type of hybrid system, both components beneficially interact to provide properties and features that they do not possess by themselves. Thus, a wide range of nanoparticles, films, and multilayered materials can result from such physicochemical interactions (Figure 3a).

#### 2.2.1. Tannin–Silica Hybrids

Researchers are consistently reporting favorable findings when developing tannin–silica systems aiming for the adsorption of metals [32,50,74]. On the one hand, silica provides excellent mechano-chemical stability, swelling resistance, and open porosity, while, on the other hand, the tannin compounds grant highly active sites [50]. Namely, the nucleophilic behavior of the C6 in the B-ring of condensed tannins is prone to form covalent bonding with electrophile species such as aldehydes [34,82,83]. Furthermore, a functionalized ceramic (e.g., amino functionalization with 3-aminopropyltriethoxysilane (APTES) [34,79,82,83]) will crosslink with tannin via glutaraldehyde, thus forming a ceramic–organic hybrid (Figure 3c). For instance, oak gall tannin can be immobilized onto mesoporous silicate particles (OGT-HMS) for degradation of direct yellow 86 dye in aqueous solution. The HMS powder was synthesized utilizing a sol-gel process and a further amination step with APTES. Finally, the tannin was cross-linked onto the functionalized mesoporous silica using glutaraldehyde as a cross-linker. The authors showed that tannin could enhance the performance of HMS towards contaminant removal [34]. This approach is also feasible when other ceramic matrices such as TiO_2_ are employed, as demonstrated by Binaeian et al. [82]. This system (TiO_2_-OGT-HMS) showed excellent photocatalytic performance towards pollutant removal when compared with bare TiO_2_ or TiO_2_-OGT systems.

Owing to their outstanding versatility, tannin-based bio-adsorbents may also be applied in more complex systems, such as core–shell structured magnetic microspheres (Figure 3b). Specifically, the magnetic core allows selective capture and recycling of magnetic carriers [84], while the ceramic layer provides a protective effect along with modifiable active sites [85]. For instance, the sorption capacity of Fe_3_O_4_@SiO_2_ microspheres towards Au^3+^ and Pd^2+^ ions was significantly enhanced by the immobilization of persimmon tannin (PT) onto the surface of silica (Fe_3_O_4_@SiO_2_@PT) [80]. The complex multiphase system comprised a Fe_3_O_4_ core, coated with a nanolayer of SiO_2_ and an external PT shell. The Fe_3_O_4_@SiO_2_@PT presented a high affinity to the noble metal ions with a very low PT loading of 1%. Besides, the different adsorption mechanisms lead to chelating or reduction of the metal ions at some specific conditions. Persimmon tannin has also been processed into a magnetic adsorbent composed of Fe_3_O_4_/PT/GO (GO, graphene oxide) for degradation of malachite green from aqueous solutions. The results demonstrated that the combination of electrostatic interaction and π-π interaction favors the performance of malachite green adsorption [27].

Furthermore, Huang et al. synthesized a tannin-based porous magnetic organic polymer (TA-MOPs) for magnetic separation of contaminants [86]. The hybrid is formed from core–shell nanoparticles (Fe_3_O_4_@SiO_2_) coated with TA forming a porous organic polymer. The large amount of phenolic -OH groups of the TA provides active sites for the rapid adsorption of contaminants from aqueous solutions, yielding high sorption capacities. Lastly, the contaminants along with the tannin-based porous magnetic organic polymer were recovered by magnetization. Similar studies were conducted demonstrating that a tannic acid nanofilm covering Fe_3_O_4_ nanoparticles can be formed by the coordination of TA with Fe^3+^ (Fe_3_O_4_@TA-Fe^3+^), exhibiting good adsorptive properties for Hg^2+^ and Pb^2+^ ions [63]. The TA-Fe^3+^ complex has also been studied as an efficient protective mesoporous nanocoating for an enzyme previously immobilized onto Fe_3_O_4_@SiO_2_ nanoparticles [87]. However, forming homogeneous TA-Fe nanofilms on curved surfaces of nanoparticles is complicated due to aggregation on the surface [88,89,90,91]. To tackle this inconvenience, Li et al. developed a layer-by-layer TA-based deposition method [77]. Herein, Fe^3+^ ions are adsorbed on the surface of Fe_3_O_4_@SiO_2_ nanospheres upon dispersion into a FeCl_3_ aqueous solution, then TA is incorporated to form the TA-Fe coordination complex. This process is repeated until a homogeneous layer is formed. Then, [Ag(NH_3_)_2_]^+^ ions are adsorbed and reduced to metallic Ag nanoparticles by the reductive capability of the TA. The resultant nanoparticles (namely, Fe_3_O_4_@SiO_2_@(TA-Fe)@Ag) were evaluated towards the degradation of 4-nitrophenol with excellent catalytic performance.

Tannins can also be useful in stimuli-responsive materials (Figure 3h). For instance, it has been found that tannin is an excellent gatekeeper in pH-responsive drug delivery systems due to its low cost, biocompatibility, steric hindrance, and as previously mentioned easiness to functionalize [79]. In this report, tannin is grafted on pre-aminated mesoporous silica nanoparticles (tannin–MSNs), by the reaction of carboxyl benzyl borate with the amino group of MSN. Subsequently, the release of rhodamine (loaded into the SiO_2_ mesopores) is blocked by the tannin coating, which is sensitive to the pH; at low pH values, the release mechanism is accelerated while the contrary effect is achieved as the pH increases. Moreover, this type of material presented enhanced biocompatibility and is a promising option for cancer therapy. The multifunctionality of tannin-based hybrids was evidenced by Wang et al. [76]. They demonstrated that different substrates coated with a TA-APTES layer can present either adsorptive, catalytic, or superhydrophobic properties by the reaction of the functional groups in the TA-APTES with octadecyltrimethoxysilane, polyethylenimine (PEI) or reducing silver ions, respectively (Figure 3d). Interestingly, their findings show that the tannin-based coatings have considerably higher performance when compared with the very well known polydopamine coatings.

#### 2.2.2. Tannin–Titania Hybrids

Overall, the development of more sustainable and cost-effective materials for several applications has allowed expanding the use of tannins in diverse fields. Their use as a building block for organic–inorganic hybrid materials have been explored with titania (TiO_2_) [82,83], zirconia (ZrO_2_) [36], and alumina (Al_2_O_3_) [92], among others [93]. As described above (Figure 2b), metal ions coordinated with tannin can be eventually reduced as the phenolic groups are oxidized, while the electrons are relocated to the complexed metal precursors. Beyond that, the coupling of tannin to TiO_2_ can be useful in photocatalytic processes. On the one hand, it can reduce the adsorbed metal ions and thereby decrease the bandgap. On the other hand, tannin enhances the reduction reaction speed and essentially acts as a shuttle for photoexcited electrons from TiO_2_ to the metal, as presented in Figure 3g. As proposed in detail by Kim et al. [78], the photoexcited electrons of tannin possess an extended lifetime because their recombination is prevented due to the unoccupied hole in the HOMO of tannin is filled by an electron stemming from TiO_2_. Photoexcited electrons can transfer from TiO_2_ to tannin, in turn leaving the electron–hole in the ceramic unpaired. These can recombine with an electron subtracted from water to form a hydroxide radical which is then scavenged by the tannin coating. Accordingly, such TiO_2_-TA hybrid can also act as a scavenging agent or harmful free radicals, as demonstrated by Son et al. [81].

Similar to the SiO_2_-based magnetic hybrid nanoparticles, core–shell TiO_2_-based materials with a magnetic component can also be useful for selective degradation of contaminants. The magnetic properties are facilitating particle recovery. Recently, Qian et al. [94] investigated the performance of TiO_2_-PEI-TA@Fe_3_O_4_ nanoparticles in the degradation of different cationic dyes. Specifically, Fe_3_O_4_ is the magnetic core, TA and PEI were employed to graft Fe_3_O_4_, and the deposited TiO_2_ nanoparticles acted as catalytic centers. Interestingly, the prepared nanocomposites presented selectivity towards the degradation of dyes with anionic or cationic nature. Such selectivity is attributed to differences in the intrinsic zeta potential of the as-prepared hybrids, which varies with the incorporation of amino groups of TA and PEI.

#### 2.2.3. Tannin–Zirconia Hybrids

Recently, tannin–zirconium hybrid materials have also been investigated regarding their coordination mechanism as well as possible applications [36,37,95]. For instance, a tannin-derived zirconium hybrid material was prepared by the rapid coordination of Zr^4+^ with tannin and, due to its high catalytic performance, functioned as an efficient catalyst for Meerwein–Ponndorf–Verley reductions, a reduction reaction type for carbonyl compounds [37]. IR-spectrometry indicated that the formation of a porous organic–inorganic network relied on the chelation between Zr^4+^ and the hydroxyl groups of the polyphenolic tannin, yielding a tannin–Zr framework. Furthermore, the network formation was suggested to be irregular resulting in an amorphous structure [37].

Moreover, similar to silica tannin hybrids, a zirconium oxide tannin hybrid has also been investigated for its usage in wastewater treatment [36]. More precisely, amino-modified zirconium salts, together with tannic acid and alginate, form a three-dimensional hydrogel by intra- as well as intermolecular hydrogen bonds. The amination of the ceramic surface was also done with APTES and follows a similar mechanism as elaborated earlier for tannin–SiO_2_ hybrids. The resultant organic–inorganic composite hydrogel allowed the adsorption of heavy metals, e.g., Pb^2+^, Hg^2+^, and CrO_4_^2−^, from wastewater. 

#### 2.2.4. Tannin-Based Metal Oxide–Carbon Hybrids

Similar to the tannin–metal hybrids, the reaction of tannins with metal oxides has also been studied regarding the generation of mesoporous carbons after hydrothermal treatment. For instance, in a study [93], it was shown that tannin can be combined with a nonionic amphiphile copolymer such as Pluronic F127 to form nanocomposites (tannin–F127). The as-prepared materials are subsequently mixed with alkaline metal acetates (Mg(OAc)_2_ and Ca(OAc)_2_) using a ball mill. In a self-assembly process, the tannins’ flavonoid units and the copolymers are crosslinked via hydrogen bonds and coordination complexes formed between the metal ions and tannin. This procedure results in a mesoporous carbon matrix with dispersed MgO and CaO NPs upon carbonization in an inert atmosphere. These hybrids exhibited high specific surface areas with selective gas sorption capacities as well as enhanced dye absorption when compared to other metal-oxide mesoporous carbons [93].

Overall, it is clear that tannin-based compounds can be reliable in both science and industrial applications. Both fields focus on the different interactions between the organic component and its inorganic counterpart. This phenomenon results in the formation of chelated complexes as well as the reduction of metal ions that can be manipulated and optimized according to their final objective. Besides their excellent anti-metal pollution properties, the usage of tannins as an eco-friendly, inexpensive, and adaptable compound is gaining increasing attention, which might bring further additional options or even substitute currently exploited recovery systems.

### 2.3. Tannin–Organic Hybrid-Like Materials

**Figure 4:**
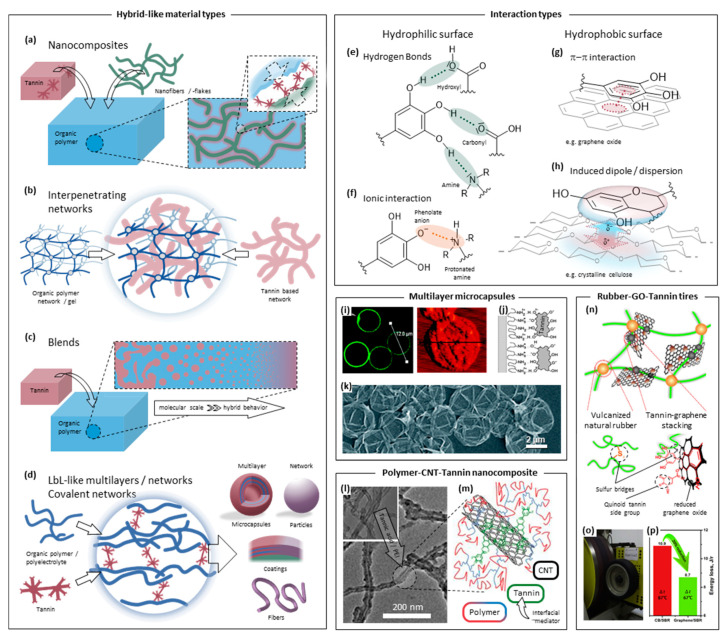
(**a**–**d**) Schematic representations and structural organization of commonly reported tannin based hybrid-like materials: (**a**) nanocomposites; (**b**) interpenetrating networks; (**c**) blends; and (**d**) physically or covalently crosslinked networks and multilayers (left) and their typical form factors (right). (**e**–**h**) Types of physical interactions of tannins with different kinds of materials and surfaces. (**i**–**p**) Examples of various types of hybrid-like materials: (**i**–**k**) Multilayer microcapsules prepared and stabilized with polyelectrolyte-LbL-like chemistry. (**i**–**j**) Adapted with permission from [96]. Copyright 2005 American Chemical Society. (**k**) Adapted with permission from [97]. Copyright 2010 Royal Society of Chemistry. (**l**,**m**) Nanocomposites based on the functionalization of pristine carbon nanotubes (inset) with tannin and polyethylenimine (**l**) and the proposed structural organization of resulting epoxy composites (**m**). Adapted with permission from [98]. Copyright 2018 Elsevier Inc. (**n**–**p**) Hybrid rubber–graphene oxide–tannin elastomer with suggested molecular organization (**n**) and its utilization to prepare and test car tires (**o**) with low energy loss (**p**). Adapted with permission from [99]. Copyright 2016 Elsevier Inc.

Similar to the interactions with metals and ceramics, tannins show versatile tendencies to interact with organic materials, such as synthetic polymers, biopolymers, or micro- and nano-sized fibers or tubes and hierarchical structures such as natural fibers. The structural organization of the resulting material can vary widely, as sketched in Figure 4a–d, ranging from intimately mixed, covalently or physically crosslinked multilayers and networks (Figure 4i–k) over polymer blends and surface functionalization (Figure 4l–m) to separate but interpenetrating networks and (nano)composites (Figure 4n–p). In particular, materials in various forms and shapes such as nanoparticles, microcapsules, coatings, or fibers have been prepared by the reaction of tannins acting as a polyelectrolyte resulting in coordination complexes with organic polymers (Figure 4d). In all instances, however, the combination of the participating materials either benefits the overall performance or leads to the emergence of novel materials with new functionalities.

#### 2.3.1. Tannin-Based Nanocomposites 

Many different reactions can be performed utilizing the reactivity of tannins to afford covalently crosslinked networks due to their multifunctionality. For example, covalent linkages can be established via the numerous nucleophilic sites or the phenolic hydroxyls. The resulting materials are typically used as glues and resins, but also as matrices for composites. Such tannin-based materials and their various properties have been comprehensively reviewed in the past [9,11,15,100]. Nevertheless, this feature, combined with the tendency of tannins to adhere to virtually any surface, makes such networks also ideal candidates for the formation of blends and nanocomposites attempting to combine, enhance, and bring forth novel properties. Numerous examples are discussed in the following which are based on reactions with polyethylenimine [101], isocyanates [102], epoxides [103], aldehydes [35], and proteinaceous compounds [104,105]. For conventional composites, many pertinent examples are known, typically employing mostly bio-based particulate or fibrous fillers but recently also more hybrid-like materials emerged based on nanoflakes [104,105], micro- and nanofibrils [103], or carbon nanotubes [98]. Frequently, compounds with disparate properties must be combined to achieve enhanced and novel properties; however, their successful fusion is often severely hampered by a lack of interfacial interaction or compatibility. In such cases, tannins can serve to interact strongly with either of the incompatible surfaces, as indicated in the zoom-in of Figure 4a and exemplified by Figure 4l,m. The versatile set of possibilities for such interactions includes hydrogen bonds, polar interactions, dispersion forces, aromatic stacking (Figure 4e–h), or metal chelation (Figure 2b).

Fibrous and nanofibrous composites and hybrids with tannins are potentially strong and lightweight materials enabling, for example, functional food packaging [106], “greener” automotive construction [107], or biomimetic air vehicles [108]. Even films without covalent interaction prepared from condensed tannin and nanocellulose possess advanced properties such as elevated chemical resistance, antioxidant properties, and UV-protection [106]. The strength of such a material can be increased by establishing stronger, ionic interactions using cationic cellulose nanofibrils instead [109]. Similar properties (i.e., antioxidant and UV-shielding) were reported alongside the enhanced strength for these films. Advantageously, tannins can often preserve their innate antimicrobial properties and confer it to the resulting material [110,111], which is frequently aimed for when developing tannin-based materials. Similarly, the metal chelation capabilities of the catecholic/pyrogallolic moieties (Figure 2b) can be preserved even when the tannin is covalently crosslinked to the surface. For example, high adsorption capacities towards Cu(II), Pb(II), and Cr(VI) were reported for biohybrid nanocellulose–tannin particles [35]. They were produced to have nanocellulose cores that were previously oxidized to the dialdehyde derivative to enable covalent functionalization with condensed tannin. Furthermore, with the introduction of covalent crosslinking also high mechanical performance nanocomposites can be prepared. This can be seen with microfibrillated cellulose and a covalent network of tannic acid and epoxidized soybean oil. Here, the presence of tannic acid was vital for crosslinking since the epoxy groups readily react with the multitude of phenolic alcohols. Thereby, tannic acid served as a curing agent and eliminated the need for a typically necessary surface treatment of cellulose to achieve a suitable dispersion in the resin [103]. Although the origins of this beneficial effect were not elaborated on, likely the phenolic moieties could attach to the hydrophobic crystalline faces of the fibrils (as sketched in Figure 4h) and enhance their solvation. Such an effect is presumably also at play in a material introduced in another study, where a hybrid made from nanocrystalline cellulose dispersed in a chitosan matrix displayed strongly elevated mechanical properties and improved water resistance when tannic acid was added. Here, the mediating action of tannic acid between cellulose and chitosan in combination with the subsequent crosslinking were responsible for the beneficial effects, which highlights its multifunctional role [112,113]. 

A similar situation is given in the preparation of biohybrid films of soy protein, cellulose nanofibrils, and graphene oxide with tannic acid. While tannic acid reduced the graphene oxide nanoflakes and attached to them via π-π interactions (as indicated in Figure 4g) as well as to the cellulose nanofibrils, presumably via dispersion forces (as in Figure 4h), it also self-polymerized. This functionalized the surfaces with a poly(tannic acid) coating and allowed intimate mixing and strong bonding to the surrounding protein matrix greatly enhancing the resultant mechanical properties [114]. A tailored load distribution via tannic acid to all members of the hybrid was identified as a major contributing factor. Here, strong mutual interaction between the different participating entities is vital, which is where the tannin plays a pivotal role. Another such example is the preparation of a high-performance hybrid material by the combination of tannic acid with graphene oxide and further with a tubular clay and natural rubber [115]. Tannic acid acted as a dispersant for graphene oxide and was responsible for the synergistic action of all participating components. In another study, highly efficient car tires were produced from a similar material, in this case based on styrene–butadiene–rubber, graphene oxide, and epigallocatechin-gallate (Figure 4n–p) [99]. The enhanced tire efficiency (Figure 4p) was evidenced by lower rolling resistance (Figure 4o) and based on the specific roles of tannin in the material. These roles were studied in detail, and the enhancement was ultimately attributed to a lower internal energy loss by diminished nano-scale friction. It was established that, by firmly attaching to the faces of the graphene oxide and via the Michael addition of polythiyl radicals (stemming from the vulcanization process), the tannin achieved a superior and partially covalent (sulfur bridges) interfacial bonding (Figure 4n). This reduced the slippage of polymer and filler with respect to each other and thus reduced internal energy loss. It is worth noting here that tannic acid again served as a reducing agent for graphene oxide, similar to the reduction of ionic metal precursors to elemental metals (as discussed in Section 2.1 and Section 2.3.3), and that this is also consistent with its frequently reported antioxidant action (also discussed at length below). In fact, tannins have been introduced as environmentally friendly and mild reducing and stabilizing agents by the same group in the past [116,117]. The reduction process seems to proceed via adjacent ether bond formation under release of water and subsequent homolytic elimination of the now quinoid tannin (Figure 4n) while a graphitic conjugate C-C bond is formed. Then, the shielding of the hydrophobic patches of (reduced) graphene oxide by the phenolic moieties of tannins attached via π-stacking (Figure 4g) is most likely the reason for the often observed enhanced dispersibility and stabilization of the graphitic flakes in solutions and in the final matrices. 

This was also convincingly presented for condensed tannin in a study, where the resulting tannin–graphene oxide complexes could be further processed into thin, flexible, and conductive nacre-like films by reaction with epoxidized natural rubber in a layer-by-layer method [118]. Similarly, the colloidal nature of such complexes, which are formed during delamination and dispersion of graphene oxide, allowed the formation of a thin film deposited on filtration membranes, which was furthermore crosslinked by reaction with polyethylenimine. This ultimately yielded membranes with improved flux, antibacterial properties, and fouling resistance [101]. Another report demonstrated the utility of these properties by enhancing the mechanical properties of graphene oxide/polyacrylamide hydrogels due to the tannin acting as a mediator, presumably enabling interactions of polyacrylamide with the faces of the graphitic flakes [119]. The interactions of tannic acid with graphene oxide even suffice to form sTable 3D networks, which can be turned into dry self-supporting monoliths [120]. Such monoliths were shown to possess electric conductivity conferred by the graphitic component as well as anti-bacterial properties owing to tannic acid. A combination of the reducing properties mentioned in the paragraph above was used to demonstrate the fabrication of hydrogels comprising tannic acid, graphene, and gold nanoparticles, which were capable of catalyzing the reduction of a model compound [121]. Tannic acid served as a dispersant for graphene oxide, as the reductant for both graphene oxide and gold ions, as well as the compound forming the connective gel network between the other components. Similar to graphene oxide flakes, tannic acid was shown to functionalize the surface of carbon nanotubes in combination with polyethylenimine (Figure 4l), forming a hybrid filler and curing material for epoxy resins [98]. The graphitic nature of carbon nanotubes makes them comparably prone to π-π interactions with the phenolic motifs of tannins as their flat counterparts, presumably resulting in the arrangement depicted in Figure 4m. This led to hybrid materials that displayed profoundly enhanced mechanical properties. On the one hand, this was attributed to the elevated dispersibility in solvents and, more importantly, in the epoxy resin. On the other hand, the authors suggested the ability of the epoxy matrix to achieve strong interfacial interaction with the carbon nanotubes via the tannin–polyethylenimine functionalization as a second factor enabling the enhancement. A similar positive effect was observed when tannic acid was added to nitrile butadiene rubber along with carbon nanotubes [122]. For instance, after the vulcanization of the rubber with carbon nanotubes but without tannic acid, the toughness was enhanced by roughly 40% while the presence of tannic acid could substantially elevate this effect to an enhancement of roughly 160%. When only tannic acid was added, the toughness was enhanced by roughly 130%; thus, these findings highlight again the key role of tannin by interacting favorably with otherwise less compatible materials. 

#### 2.3.2. Interpenetrating Networks and Polymer Blends

As illustrated by the above example and in contrast to other nanocomposites discussed so far, enhanced mechanical properties are also commonly observed for materials devoid of fillers such as tubes, fibers, or flakes. For instance, mechanically improved elastomers were demonstrated by utilizing the ability of tannic acid to form a multitude of physical bonds with natural rubber. This was achieved by establishing a network in an interpenetrating fashion (Figure 4b), formed from tannic acid during vulcanization within the rubber which homogeneously stabilized the material [123]. The toughness and strength were enhanced by up to 190% and 200%, respectively (comparable to commercial fillers at much higher loadings). The results discussed in the previous paragraph (see [122]) are in quite good agreement with this, although the formed interpenetrating network seems to be even more favorable than uncontrolled blending. A different, quite outstanding approach towards such a material utilizes enzyme-mimetic catalytic crosslinking of condensed tannin which can yield flexible hydrogel networks of various properties. However, these networks could be established in an interpenetrating fashion to afford nanocomposites with natural rubber, again exhibiting enhanced mechanical properties [124]. Similarly, flexural properties could be enhanced in bio-based nanocomposites comprising poly(lactic acid) (PLA) and a polyurethane network which was formed from propylene oxide derivatized condensed tannin by reaction with polymethyldiisocyanide [125]. On the other hand, no notable beneficial or adverse effects on thermomechanical properties could be documented for the use of quebracho tannin as a micrometer scale filler material in composite injection-molded PLA parts [126]. The findings of the studies outlined above all go in line with the notion that the most beneficial and hybrid-like effects emerge for materials mixed at molecular length-scales, as indicated in Figure 4c.

**Figure 5:**
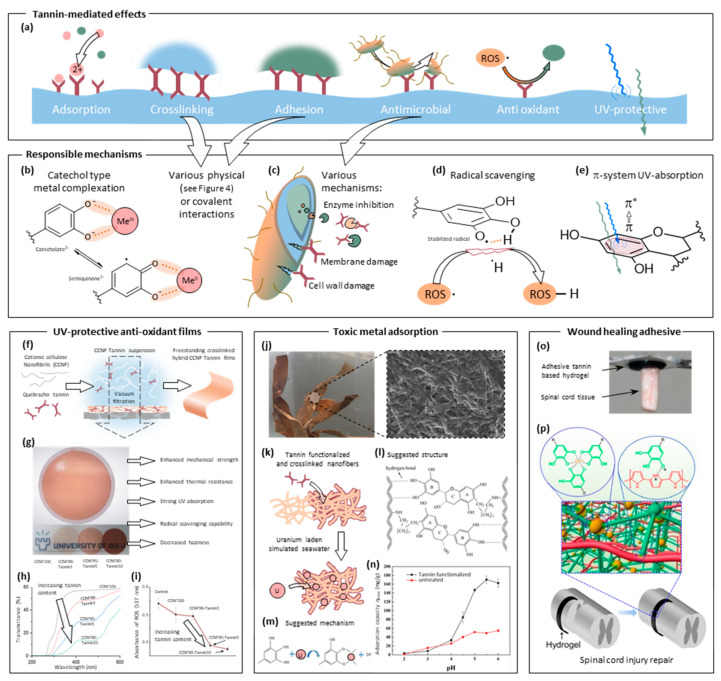
(**a**) Schematic representation of various possible functionalities accessible with tannin-based hybrid materials. (**b**–**e**) General schematic representations and mechanisms of valence tautomerism between the catecholate-metal(III) and semiquinone-metal(II) isomers (**b**); several known points of antimicrobial action of tannins (**c**); reactive oxygen species (ROS) scavenging mediated by phenolic homolysis (**d**); and UV-shielding by absorption due to π-π* transitions of the phenolic aromatic rings (**e**). (**f**–**i**) Schematic representation of fabrication (**f**); optical appearance and key features (**g**); UV–Vis spectra (**h**); and ROS scavenging ability (**i**) of cellulose–tannin films. Adapted with permission from [109]. Copyright 2019 Elsevier Inc. (**j**–**n**) Optical appearance and microstructure (**j**) and schematic representation (**k**) of gelatin nanofiber–tannin based adsorbent material tested with Uranium ions (**k**) with suggested chemical structure (**l**) as well as the complexation mechanism (**m**) and the resultant adsorption isotherms (**n**). Adapted from with permission [132]. Copyright 2018 Elsevier Inc. (**o**,**p**) Adhesive polypyrrole–tannin–Fe(III)-based hydrogel for spinal cord injury wound healing applications with high conductivity and cytocompatibility. Adapted with permission from [133]. Copyright 2018 American Chemical Society.

In addition to mechanical enhancement, blending tannins into polymers can also serve the purpose to equip them with additional functionalities, some of which are sketched in Figure 5a. For example, the resulting materials can be imparted with resistance to oxidative species by the radical scavenging nature of tannins and with greatly enhanced UV-resistance (Figure 5d,e). The phenolic hydroxyl groups of tannins can release a hydrogen radical (via homolysis), which can then react with and neutralize other radicals such as reactive oxygen species (ROS) (Figure 5d). The radical phenolic ring, which is stabilized by its adjacent hydrogens or delocalization, is thereby turned into a semiquinoid (for structural reference, see Figure 5b) and, after another homolysis step, into a quinoid ring. This radical scavenging capability is often preserved, for instance in multilayered films of tannic acid and polyelectrolytes, as demonstrated by photometric monitoring of a sample radical species [127]. A similar effect was demonstrated with condensed tannin from larch bark and poly(vinyl alcohol), where pronounced antioxidant properties were revealed in solvent cast membranes [128]. Similarly produced films of ethyl cellulose loaded with small amounts of grape tannin also exhibited antioxidant properties [129]. A combination of several beneficial effects was observed when quebracho tannin (condensed) was used together with modified cellulose carrying cationic groups in order to establish biohybrid nanofilms via simple vacuum filtration (Figure 5f,g). Here, interactions of (partial) negative charges of tannin with the introduced positive charges on cellulose nanofibrils (akin to those in Figure 4f) were sufficient to render the tannin insoluble, to physically crosslink the formed films and thereby impart them with enhanced mechanical, UV absorbing (Figure 5h) and antioxidant properties (Figure 5i) [109]. The UV-shielding capabilities of tannins stem from the absorption maximum of the aromatic ring π-π* transitions (Figure 5e), which are redshifted into the UV-B region by the presence of the hydroxyls [6]. Thus, this property is, although often of minor interest, almost ubiquitously present in tannin modified materials to a certain extent and yet sometimes of primary interest, for example for UV-protective packaging [128]. Many chemical modifications of tannins do not substantially change these UV-shielding properties. Therefore, condensed mimosa tannin could be prepolymerized with hexamine and used to prepare blends with polypropylene and polymethylmethacrylate displaying preserved resistance to UV exposure and largely unaffected material strength [130]. In another study, condensed tannin was modified with aliphatic residues in order to increase compatibility with different bio-based polymers. Again, the polymer blends showed pronounced UV-resistance at largely unaffected mechanical properties [131].

Although sometimes negligible effects on the mechanical properties are observed when blending tannins with other polymers (at least when covalent crosslinking is absent), some studies point towards at least morphological changes. For instance, in a systematic study [134], the effect of tannic acid on the crystallization behavior of various polymers, due to strong physical interactions, was presented and for polyethylene glycol in terms of the resultant dendritic morphology of spherulites at different tannic acid loadings [135]. A shift in polymer morphology of chitosan nanocomposited with tannic acid and nanocrystalline cellulose was also reported [112]. Others investigated the influence of an interaction with tannic acid in solution. For instance, it has been shown that TA can alter the physical behavior of a polymer such as the lower critical solution temperature of poly(*N*-isopropylacrylamide) [136]. Such a behavior—rendering polymers insoluble by physical interaction—was used to prepare nanofibers from usually well soluble poly(N-vinyl-caprolactam) suitable for tissue engineering applications in an aqueous environment and could therefore serve as valuable scaffolds [137]. The polymer properties can also be changed by only functionalizing the materials, for instance when tannins are attached to the surface. An early example demonstrating this possibility of strong interactions between tannic acid and polymeric surfaces was a study focusing on the effects of the adsorption of tannic acid to an acrylonitrile/vinyl-acetate co-polymer on the surface free energy [138]. The authors concluded that the interaction was primarily governed by hydrogen bonding and dispersion (Lifshitz–van der Waals) forces rather than electrostatic interactions. 

#### 2.3.3. Polyelectrolyte Complexes, LbL-like Materials, and Coatings

Similar physical hydrogen bonding and hydrophobic interactions (as described in the study above) between gelatin and tannins can suffice to induce coacervation. Accordingly, hydrolyzable tannins could be used to assemble nanospheres in a quite narrow range of concentrations affording the formation of a stable suspension [139]. Such nanospheres are potential candidates for encapsulation and therefore biomedical applications. On the other hand, this interaction strength induces the adsorption of tannins to the surface of collagen fibers in an aqueous environment and subsequently the adsorbed layer can be covalently crosslinked to the proteinaceous fibers by treatment with glutaraldehyde. This was demonstrated, for instance, with the complex tannin epigallocatechin-3-gallate [140] as well as condensed bayberry tannin [64,141]. Fibers functionalized with the latter were shown to adsorb toxic Hg^2+^ ions, thereby acting as a cheap bio-based adsorbent material (Figure 5a) due to their metal complexation behavior (Figure 5b) [64]. Similarly, electrospun nanofiber membranes made from a blend of gelatin (i.e., collagen) and poly(vinyl alcohol) were hybridized with a tannin coating (Figure 5j,k). This enhanced mechanical properties due to crosslinking (Figure 5l) and equipped them with high adsorption capacity for uranium in simulated seawater (Figure 5k,m,n) [132]. Another interesting use of the tannin layer is made by generating noble metal nanoparticles on their surface by in-situ metal ion reduction. This reduction functionality might proceed through a combination of the reaction sequence outlined in Figure 3c and the metal-chelate valence tautomerism depicted in Figure 5b. Both processes lead to the oxidation of tannin and an eventual reduction of the ions to the elemental metal. Examples include gold [141], silver [142], palladium [140], and even core–shell silver–nickel particles [143], where the resultant nanocomposite materials were used, for instance, as heterogeneous catalysts or for microwave radiation absorption. 

In pure collagen networks, non-covalently attached tannic acid was also shown to increase the stability against enzymatic degradation by inactivation (Figure 5c) and to have positive effects on wound closure and healing [144]. This further indicates that the antimicrobial properties can be preserved and utilized for biomedical applications. Accordingly, the adhesive properties along with the microbicidal action of tannic acid were used to prepare antimicrobial tissue adhesives by grafting tannic acid onto gelatin and subsequently crosslinking the modified biopolymer into an adhesive gel with silver nitrate [145]. 

Since the hydroxyls of tannic acid can act as H-bond donors for H-bond accepting amine groups (Figure 4e), they enable it to function as a mediator and physical crosslinking agent between polymers carrying amine groups. In the case of collagen and chitosan, such an H-bond network enhances the mechanical and chemical properties of their blends [146]. This is necessary to efficiently construct scaffolds for tissue engineering, although their performance towards cell proliferation or cytotoxicity has not been investigated. However, as discussed below, pertinent biomedical materials tend to perform well in this regard and typically strongly benefit from the presence of tannins. For example, a potential nanofibrous wound dressing material based on tannic acid, chitosan, and pullulan was prepared by force spinning their mixture in water. Subsequent crosslinking was afforded by citric acid under elevated temperature, and these fibers exhibited favorable properties in terms of cell migration and adhesion [147]. Furthermore, the water resistance and swelling behavior can be enhanced by virtue of such abundant physical interactions, exemplified by the utilization of tannic acid in nanocomposites comprising chitosan and chitin nanofibrils [148]. In this material, at high nanofibril loading, tannic acid acted as a plasticizer enhancing the possible elongation at break leaving the tensile strength largely unaffected. However, it seems that the conformational arrangement of chitosan can be somehow disturbed by the presence of tannic acid also leading to a change in polymer morphology. Nevertheless, the film formation capabilities and enhanced mechanical properties make such materials quite attractive for different fields. 

The metal complexation capability conferred by the incorporation of tannins as described above for collagen was also explored with chitosan, highlighting the generic nature of the approach. For instance, condensed persimmon tannin was covalently crosslinked via glutaraldehyde onto chitosan after a tannin layer had been established via physical interaction on the surface. This yielded a cheap, almost entirely bio-based hybrid material allowing the efficient and selective adsorption of Pd^2+^ from wastewater mixtures [149]. Another work reported a hybrid material based on the interaction and subsequent crosslinking of hydrolyzable chestnut extract with chitosan and inorganic sericite via glutaraldehyde, which was processed into microcapsules [150]. These microcapsules were then characterized towards the use as adsorbent materials for toxic metal ions contained in wastewaters, evidencing the intact metal complexation functionality of the catechol groups. However, the use of such microcapsules based on the interaction of tannic acid and chitosan is not limited to metal adsorption purposes. They were also demonstrated to enable the pH-responsive encapsulation and release of a model protein (bovine serum albumin) [151]. The incorporation of tannic acid into such capsules was further shown to have a potential protective effect towards encapsulated substances which might be sensitive to oxidizing agents [127].

The same group, Lvov et al., initially introduced this possibility to assemble thin films and hollow capsules (Figure 4i) by virtue of physical interactions between tannins and polymers (as sketched in Figure 4d) in a landmark study utilizing a layer-by-layer technique [96]. Two different cationic polyelectrolytes, poly(dimethyldiallylamide) and poly(allylamine), were demonstrated to interact intimately with tannic acid (Figure 4j), and the stability of the layer-by-layer assembled materials was found to be highly pH-dependent. As discussed above, such multilayers were later shown to additionally possess pronounced radical scavenging capabilities [127]. Later on, several different nonionic polymers were studied towards their ability to form layer-by-layer structures with tannic acid, highlighting once again the pH-dependent and tunable nature and, thus, their potential for biomedical applications [152]. Even capsules based on the physical crosslinking of tannic acid with protein (bovine serum albumin) were demonstrated to afford enzymatically degradable multilayered vessels for medical applications [153]. As an example, the stimuli-responsive release of drugs from multilayers of tannic acid and micelles of a block copolymer (poly(ethylene glycol)-b-poly(2-hydroxylethyl methacrylate)) carrying an entrapped cancer drug was demonstrated [154]. On the other hand, microcapsules have been prepared from tannic acid and several different, more hydrophobic polymers, which were used to act as microreactors (Figure 4k) [97]. Remarkably, these capsules were stable in a wide range of pH values besides possessing a certain amount of responsiveness. With one of these combinations, tannic acid with poly(N-vinylpyrrolidone), the generation of nanoparticles carrying a model anti-cancer drug was recently demonstrated which exhibited promising features for oral chemotherapy [155]. Alternatively utilizing ionic interactions, Tanfloc (an amine-functionalized high molecular weight polyelectrolyte based on polymerized condensed tannin trademarked by Tanac S.A.) can be used in the preparation of biocompatible structures and coatings. The partial positive charges of the amino groups aid in the formation of polyelectrolyte complexes with, for instance, alginate [156] or glucosaminoglycans [157] by interaction with their negative moieties to generate materials that are cytocompatible [156] and even promote stem cell adhesion and proliferation on different substrates [157]. These properties are ideal for tissue engineering scaffolds. Such scaffolds have been prepared as non-woven, water-resistant fiber membranes by electrospinning solutions of Tanfloc mixed with poly(ε-caprolactone) [158]. The suitability of tannin for tissue engineering scaffolds has furthermore been demonstrated with a hydrogel based on hyaluronic acid and polyethylene glycol. The mechanical properties, the chemical, and enzymatic resistance as well as the antioxidant potential could be significantly enhanced by the action of tannic acid [159].

The multitude of physical interactions in which tannic acid can participate makes it a valuable precursor candidate for adhesive materials in most challenging environments and substrates. Mussel inspired adhesives have drawn considerable interest in the past decades [160] (and continue to do so [161]) as a class of materials where the ability of catechol groups to mediate complex interactions with the surface is key. Tannins, especially tannic acid has been demonstrated to be useful in the preparation of such adhesive hydrogels. This is because of the diverse possibilities of covalent and non-covalent interaction, which are akin to those of L-DOPA found in mussel adhesives. For example, a tissue- and mucoadhesive material based on tannic acid and poly(ethylene glycol) was devised by forming and subsequently drying a hydrogel established by simply mixing diluted solutions of both components [162,163]. Stretchable hydrogels based on a hydrogen-bond network between tannic acid and DNA with adhesiveness to biological tissue were shown to display hemostatic ability making such gels potential candidates for biomedical applications such as wound healing glue [164]. Therefore, they were shown to exhibit good adhesion to mucin-lined surfaces ex vivo as well as in vivo. Similarly, the adhesive properties along with the microbicidal action of tannic acid were used to prepare antimicrobial tissue adhesives by grafting tannic acid onto gelatin and subsequently crosslinking the modified biopolymer into an adhesive gel with silver nitrate [145]. The developed glue showed good adhesion strength as well as excellent cytocompatibility and antimicrobial properties. As already discussed at length in the above sections, metal complexation can be used to form stable metal–phenolic networks. Therefore, hydrogels can be expanded by this possibility via the introduction of tannic acid. For example, in combination with poly(allylamine) and trivalent ions physically and ionically crosslinked gels were prepared, which also underwent a certain degree of covalent crosslinking upon increase of pH. The gels had roughly 1% of the tensile strength of cotton fibers while being pH responsive [165]. A metal–phenolic network was also used for the formation of a composite hydrogel with carboxylated agarose, where interaction of the two components was mediated by the addition of zinc ions [166]. The generic nature of this approach was further demonstrated by a systematic study revealing the available parameter space for hydrogel formation for various polymers with subsequent crosslinking by Fe(III) ions [167]. Such hydrogels typically displayed pH-responsive stability and were shown to be potential candidates as antibacterial and anti-inflammatory wound healing materials [166]. Recently, conductive hydrogels for the application in wound healing of spinal cord injuries were developed, where tannic acid played crucial mechanical, physical as well as physiological and antimicrobial roles (Figure 5o) [133]. The conductive polymer polypyrrole was crosslinked and doped by the presence of tannic acid, which in turn was further cross-linked by the addition of Fe(III) ions (Figure 5p), making it possible to tune the mechanical properties of the resulting gels. The combination of the physical and chemical properties of the resultant gels induced significant beneficial effects for the wound healing of spinal cord injuries in vivo.

In summary, the use of tannins in combination with other organic, mostly polymeric, precursors to enable advanced hybrid-like materials has been reported frequently in the recent past. The versatile nature of the phenolic molecules in terms of the possible interactions and reactions leads to a multitude of options where tannins can play successful and beneficial roles. Undoubtedly, two major directions that are being explored heavily at the moment are the successful fusion of graphitic materials with polymers of little compatibility afforded by the presence of tannins and the utilization of tannins in biomedical materials. 

## 3. Emerging Technological Opportunities 

The exceptional potential of tannins as adsorbent materials, in the form of tannin-derived hybrid materials (tannin–metal, tannin–ceramic, and tannin–polymer hybrids), or biomedical applications, such as drug-delivery agents (microcapsules), scaffolds for tissue engineering, or hydrogels for wound healing, have been recognized during the last years and first applications were successfully tested or are already established. Nevertheless, during the past decade, intelligent material systems have received increasing attention because they exhibit some novel performances, such as sensing, adaptive responses, or shape memory capabilities. Such intelligent systems do not consist of a single material, rather they are hybrid composites, and are assembled by methods such as 3D or 4D printing and/or self-folding mechanisms.

### Stimuli-Responsive, Shape-Changing Materials and 4D Printing

**Figure 6:**
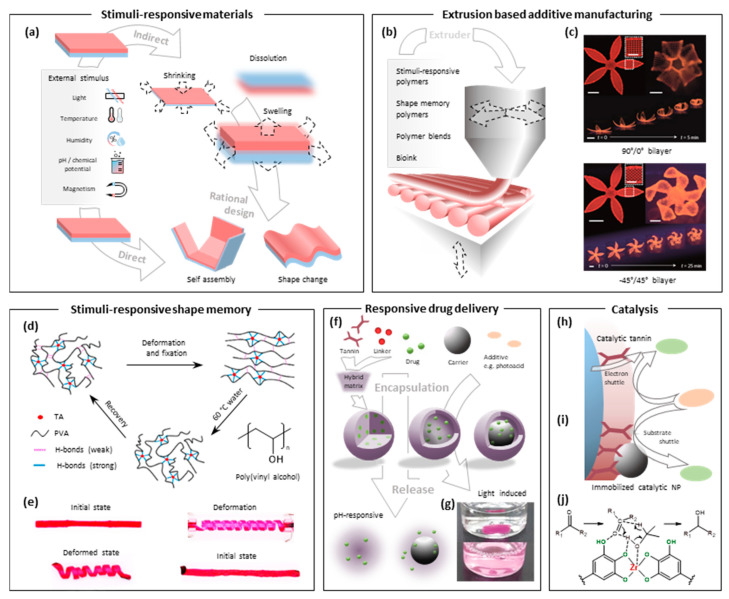
(**a**) Schematic representation of different types of stimuli and responses like swelling, shrinking and dissolution. Responses can directly result in complex shape change and self-assembly or be indirectly constructed by rational design. (**b**) Schematic representation of extrusion-based additive manufacturing (EAM) showing how material is deposited in layers of individual strings. Various types of materials can be processed and accurately arranged in space. (**c**) Example of self-assembling structures obtained via rational design elaborated by EAM. The behavior can be tailored by changing the alignment of the individual layers in the bilayer structure. Adapted with permission from [176]. Copyright 2016 Springer Nature. (**d**,**e**) PVA hydrogels with shape memory properties obtained by the addition of tannic acid. (**d**) Proposed structural changes where H-bonding between individual PVA strands is weak and easily broken during deformation and reformed for the fixation of the transient shape while TA forms strong, unchangeable crosslinks between PVA strands providing recovery and shape memory. (**e**) Optical photographs of the PVA-TA hydrogels during a cycle as sketched in (**d**). Adapted with permission from Ref. [175]. Copyright 2016 American Chemical Society. (**f**) Strategies for the formulation of tannin-based stimuli-responsive drug delivery materials. Tannin-based matrices can encapsulate drugs within nanospheres, microcapsules, or particulate carriers. The matrices are suited for pH-responsive release and can be modulated by additives to elaborate other responses such as (**g**) UV-light induced release. Adapted with permission from [70]. Copyright 2017 Wiley-VCH. (**h**,**i**) Schematic representation of roles that tannins play in hybrids for catalytic applications where it was shown to effectively shuttle electrons from a photocatalytic carrier to the substrate [77] (**h**) or enhance catalytic performance by increasing substrate availability by localized adsorption [76,94](**i**). (**j**) Proposed catalytic mechanism of a hybrid tannin–zirconium network for a type of carbonyl reduction reactions [37].

Shape-morphing systems can be found in many areas, including automotive industries, smart textiles, autonomous and soft robotics, tissue engineering, or (bio-)medical devices. To solve complex problems, nature has evolved a large number of soft materials that can alter their properties as well as their geometry in response to external stimuli and thereby fulfill specific tasks (Figure 6a) [168,169,170]. Therefore, biomimetic materials attempting to reproduce such behavior are actively researched by utilizing advanced materials such as shape memory polymers (SMPs) [170]. SMPs possess interesting properties; for example, they require small actuation forces, possess variable stiffness, and can reversibly sustain large deformations [171]. They are also programmable insofar as a defined stimulus causes the material to react automatically in a predetermined way [172,173]. Thus, SMPs have, in principle, intrinsic actuating, controlling, and sensing capabilities encoded in their structure and chemistry so that they can directly afford shape-changing and self-assembling objects (direct route in Figure 6a). Poly(vinyl alcohol) (PVA) hydrogels were the first reported shape memory hydrogels, where the shape memory was afforded by covalent crosslinking with glutaraldehyde [174]. Recently, Chen et al. replaced the aldehyde with tannic acid and with the consequential formation of linkages based on multiple strong H-bonds between TA and PVA, these hydrogels could attain excellent mechanical and shape memory properties (Figure 6e–f) [175]. Moreover, tannic acid inhibits PVA crystallization, which, along with the amorphous nature and strong bonding, results in excellent mechanical properties. The stronger PVA-TA bonds act as “permanent” linkages, while the weaker H-bonds between individual PVA chains act as reversible linkages (Figure 6d). The reversible nature of the latter imparts the PVA−TA hydrogels with fast shape memory properties (Figure 6e). These hydrogels could be directly utilized to elaborate shape-changing or self-assembling objects, as illustrated in Figure 6a. 

On the other hand, such objects can be constructed by rational design and appropriate spatial arrangement of less “smart” responsive parts (indirect route in Figure 6a). In this respect, extrusion-based additive manufacturing (EAM) methodologies are ideal for fabricating complex objects with good spatial control (Figure 6b). In recent years, the complexity of objects has been extended significantly even to highly intricate objects such as functional organs and biomimetic shape-morphing structures (Figure 6c,d) [176]. In the latter, complex shape changes occur over time which are actuated by localized swelling anisotropies introducing time as the fourth dimension [176]. Most 4D printing approaches use EAM techniques to establish the necessary spatial arrangement of responsive parts and sections. Such responsive materials utilizing tannin-based hybrid like materials have been developed in the recent past. For example, Liao et al. [177] reported the successful EAM processing of a blend of PLA and acetylated condensed tannin (AT) and studied the effect of AT loading on polymer crystallinity, tensile properties, water absorption as well as degradation rate. PLA/AT blends with higher AT loading levels showed significantly higher water absorption and a decrease in crystallinity, which is favorable for faster degradation during exposure to the aqueous (especially alkaline) environment. Compounds such as these PLA/AT blends are interesting for medical applications, e.g., quickly dissolving implants or the release of therapeutic tannins and drugs, as well as swelling (fast) or sacrificial (slow) parts of shape-changing objects. Water- and pH-responsive hydrogel threads changing their mechanical behavior upon exposure are another example [165]. The employed polyallylamine/TA/Fe(III) network could be crosslinked to change elastic modulus at high pH, and it changed tensile strength with humidity, potentially enabling triggered response systems. A specific use case of a similar system has been demonstrated with pH-responsive, tannin-based, antibacterial wound dressings [166]. Advanced wound dressings have become increasingly popular in recent years typically using silver-coated or nanoparticle impregnated dressings, but replacing them by the use of TA with carboxylated agarose and zinc ions for crosslinking provides a promising platform. Hydrogel scaffolds for the pH-controlled release of TA were produced and found to display antibacterial and anti-inflammatory properties as well as a lack of cytotoxicity in simulated wound assays. 

Such biomimetic materials based on tannins seem promising not least due to their non-toxic properties for mammalian cells. The sensitivity to ambient conditions and reversibility can also lead to tannin-derived hybrid materials that respond to certain stimuli in deliberately chosen ways and allow the rational design of objects that address so far unmet challenges. Tailored changes in their properties and the geometry suggest dynamically adapting materials that meet the complex needs of tissue healing and regeneration processes. It also suggests the utilization in innovative biomedical applications using their stimuli-responsive nature for smart drug delivery materials [164].

As noted throughout this review, the intrinsic properties of tannins greatly benefit them, which is reflected by the large number of applications that they have in various areas of both science and technology. Recently, this has been accentuated by mixing them with compounds of different nature such as metallic elements, ceramic matrices, and organic species. As a result of these combinations, ranging from a molecular level to a macroscopic product, the variety of functionalities of these materials has been further increased. 

They are not only useful in well-known systems, such as the recovery of metal ions in aqueous effluents, the synthesis of materials, or in the mechanical reinforcement of organic matrices, but tannins are also finding their place in barely explored applications such as photocatalysis, advanced drug delivery systems, in adjustable morphing materials, or UV-shielding. Even in cutting-edge technologies, it shows very beneficial characteristics by the addition of tunable chemical bonds resulting in shape-memory materials, or 3D and 4D printing inks. In fact, in the case of drug delivery various different routes have been taken to utilize their pH-responsive properties in the form of nanospheres, microcapsules or coatings on particulate and porous drug carriers (Figure 6f). By the addition of modifiers, other stimuli have also been demonstrated to be accessible such as UV-light responsive drug release by incorporation of photodegradable acids (Figure 6g) [70]. Tannins have also been shown to play various roles in catalytic applications (Figure 6h–j). Interestingly, they have been proposed to be amenable to photocatalysis, where they transfer and effectively shuttle photoexcited electrons to substrates from TiO_2_ (Figure 6h) [77] or they play active roles in catalytic cycles (Figure 6j) [37]. In addition, the preferred coordination of substrates near active sites (Figure 6i), as seen for tannin-immobilized catalytic nanoparticles, has been shown to significantly enhance catalytic activity [76,94].

A clear trend can be found with respect to tannin-based hybrid materials, in which the value provided by the tannin compound is of utmost importance since in some cases it facilitates the synthetic protocol, but at the same time provides new functionalities. All this together favors the extrapolation towards new fields of application.

## 4. Conclusions and Future Perspectives

Tannins are bio-sourced molecules that can be obtained from different plants, and their unique combination of physical as well as chemical interactions, their diverse structural variants, and the ease of processing have led to the increased interest of researchers and industry peers in recent years. This is reflected by the substantial amount of progress made by developing tannin-based materials as an alternative to current solutions by achieving similar, enhanced, or even extended performances with the benefit of the “bio” suffix and its implications. Therefore, owing to their chemical structure and versatility, tannins have found applications in a wide variety of fields as they can form physical or chemical interactions with compounds possessing most different characteristics. Derived from this interesting capacity, tannin-hybrid materials such as metal adsorbents, core–shell nanostructured materials, smart materials, and catalytic compounds, to name a few, have been developed, trying to overcome the difficulties that each separate constituent cannot overcome by itself. Significantly, the choice of the inorganic matrix of tannin-hybrid composites is open not only to a diverse range of ceramics such as silica, titania, or iron oxides but also to several metallic species, thus broadening their potential fields of application. Moreover, the integrated tannin compound in any of its shapes (i.e., intermediate layer, the active compound, reducing agent, etc.), provides the final product with enhanced chemical activity and multifunctionality in the same system, making tannins potential candidates for comprehensive studies at different levels. 

Considering the importance of microstructural aspects for ceramics and how this might be reflected in the final product, deeper studies must be conducted to investigate how these fundamental variations might influence the response of the hybrids towards particular interactions and working environments. Furthermore, the structural variety of tannins will add yet another level of diversity for each respective ceramic. Thus, to gain a more complete understanding of these as well as potential hybrid systems, fundamental knowledge of the factors that lead to specific outcomes must be obtained by conducting additional comprehensive investigations. 

As demonstrated by the reviewed literature, the combination of a tannin-based compound with another organic component that displays considerably different characteristics commonly results in interesting and very favorable functionalities. Such features allow the classification of the resulting materials as hybrid-like systems. Clear examples are the formulation of complex hydrogel networks or tannin-based nanocomposites, attempting to diminish poor responses towards certain stimuli such as mechanical stress and simultaneously add functionality such as UV-shielding, antimicrobial, or responsive behavior.

Lastly, it is evident that there is a profound knowledge base regarding the useful characteristics and adaptable properties of tannins performing in hybrid nanostructured materials. Therefore, its wide-open fields of application will be easily broadened even more by innovative combinations and use along with the most promising yet to exploit areas. However, it is also clear that there are voids in the research that need to be addressed in order to expand the possibilities of tannin-hybrid derived materials based on rational design, as has already been seen in some examples.
